# MicroRNAs in Neurotoxicity

**DOI:** 10.1155/2012/870150

**Published:** 2012-03-06

**Authors:** Prameet Kaur, Arunmozhiarasi Armugam, Kandiah Jeyaseelan

**Affiliations:** Department of Biochemistry, Yong Loo Lin School of Medicine, National University Health System, National University of Singapore, 8 Medical Drive, Singapore 117597

## Abstract

MicroRNAs are gaining importance as regulators of gene expression with the capability to fine-tune and modulate cellular events. The complex network with their selective targets (mRNAs/genes) pave way for regulation of many physiological processes. Dysregulation of normal neuronal activities could result in accumulation of substances that are detrimental to neuronal functions and subsequently result in neurotoxicity. Neurotoxicity-mediated pathophysiological conditions could then manifest as diseases or disabilities like Parkinson's and Alzheimer's which have debilitating implications. Such toxicity can be a result of individuals predisposed due to genetic inheritance or from other sources such as brain tumours. Neurotoxicity can also be brought about by external agents like drugs and alcohol as well as brain injury with miRNAs playing a pivotal role in diseases. It is therefore vital to understand the expression of these microRNAs and their impact on neuronal activities. In this paper, we discuss some of the neuronal pathophysiological conditions that could be caused by dysregulated microRNAs.

## 1. Introduction

MicroRNAs (miRNAs) are fast emerging as important regulators of gene expression, controlling almost every activity of a cell from development to cell death [1–6]. These riboregulators were first discovered in *Caenorhabditis elegans* in 1993 [7] after which numerous reports on the miRNA-mRNA relationships and the resulting functional regulations have been documented [6, 8–14].

MicroRNAs (miRNAs) are endogenous, small (~23 nt), noncoding RNAs that are capable of regulating translation and transcription of specific mRNAs and gene promoters [7, 15–19]. A single miRNA is also capable of regulating a myriad of genes [20]. These miRNAs are derived from long stem-loop transcripts by the action of nucleases Drosha and Dicer (RNaseIII enzyme). The mature miRNA forms a complex with the RNA-induced silencing complex (RISC) and subsequently interacts with its targets to bring about RNA interference (inhibition or activation) [21].

Tissue-specific and organ-specific miRNAs have also been elucidated [22]. MiRNAs are abundant in the central nervous system. MiRNAs that are specifically expressed and enriched in the brain are implicated in maintaining normal neuronal function and homeostasis which in turn is associated with memory, neuronal differentiation, synaptic plasticity, and neurogenesis as well as neuronal degeneration [23–26]. The brain-specific miR-9 targets the *stathmin* mRNA and has been implicated in early neurogenesis and proliferation while decreasing migration of young neural progenitor cells [27]. Another brain-specific miRNA, miR-124 has been implicated in neuronal differentiation. MiR-124 directly targets the *polypyrimidine tract-binding protein 1* (*PTBP1*), which encodes a global repressor of alternative pre-mRNA splicing in nonneuronal cells. MiR-124 mediated reduction in *PTBP1* levels, increases the correctly spliced PTBP2 protein which promotes nonnervous system to nervous system-specific alternative splicing patterns. Hence, miR-124 promotes nervous system development as well as plays a key role in the differentiation of progenitor cells to mature neurons [28]. The brain-specific miRNAs could individually or collectively promote and maintain neuronal development [29]. Numerous other miRNAs have also been reported in brain function including miR-134 which modulates spine and dendrite development [4].

Neurons usually require a tight control in several gene expression pathways. Dysregulation in any one of these could have drastic effects on the expression of downstream genes and proteins that could eventually offset the balance and function of the neurons. Abnormal expression and protein function could give rise to the inability of the neuron to clear waste products. Accumulation of waste products could result in toxicity, cell death, or malfunction of the neurons.

Neurotoxicity is a result of the adverse effects of chemical, biological, and certain physical agents on the nervous system and/or behavior during development and in maturity. These agents could be either endogenously produced by the nervous system or could be acquired from exogenous sources. Both the central and peripheral nervous system are very sensitive, such that any minor change in the structure or function of the nervous system might have profound consequences on neurological, behavioural, and related body functions [9]. This applies to miRNA levels as well. The cells of central nervous system (CNS) comprise neurons and glial cells (astrocytes, oligodendrocytes) while the peripheral nervous system system has mainly Schwann cells [30].

Ablation of miRNA processing enzyme, *Dicer* has been found to result in cell death, and ataxia in postmitotic Purkinge cells [31]. Similarly, Tao et al. [32] reported that Dicer is essential for maturation and maintenance of cerebellar astrocytes. Dysfunctional dicer has been found to result in neurological disorders like ataxia, seizures, severe progressive cerebellar degeneration and premature death [32]. These events could also lead to the spread of neurotoxicity to the surrounding neurons which depend on neurotransmitters like glutamate and acetylcholine [33].

**Figure 1:**
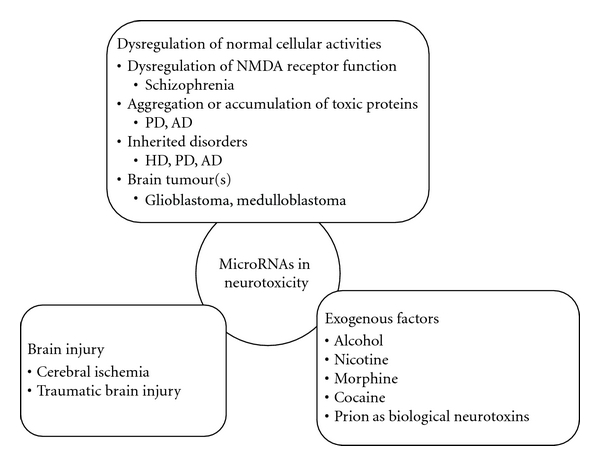
Overview of agents that confer neurotoxicity in the nervous system with some examples of diseases. Abbreviations: AD: Alzheimer's disease; HD: Huntington's disease; NMDA: N-methyl-D-aspartate; PD: Parkinson's disease.

MiRNAs are therefore of utmost importance in maintaining neuronal homeostasis and their dysregulation could result in neurotoxicity. This review will focus on miRNAs that have been demonstrated to contribute to different types of neurotoxicity (Figure 1).

## 2. Dysregulation of Cellular Activites That Lead to Neurotoxicity

Malfunction of the cellular machinery could lead to alteration of miRNA expression which would result in aberrant expression of target mRNAs. This dysregulation could alter several downstream pathways and manifest effects like deficiency in clearance of cellular by-products. These alterations in miRNA expression and subsequent accumulation of neuron specific by-products are responsible for age-dependent neurodegeneration [5].

### 2.1. Dysregulation of NMDA Receptor Function

N-Methyl-D-aspartate (NMDA) receptors are responsible for neurotransmission as well as neuronal plasticity [34]. Dysregulation of gene expression in this pathway has been shown to give rise to disorders like schizophrenia [35], bipolar disorder [36] and autism [37, 38]. Disruption of NMDA-mediated glutamate signaling has also been linked to behavioral deficits displayed in psychiatric disorders such as schizophrenia. Kocerh et al. [39] showed that pharmacological (dizocilpine administration) or genetic (*NR1* hypomorphism) disruption of NMDA receptor signaling reduced the levels of a brain-specific miRNA, miR-219, in the prefrontal cortex (PFC) of schizophrenic mice models. MiR-219 has been shown to negatively regulate a vital molecule, *Ca^2+^/calmodulin-dependent kinase II *γ** (*CaMKII*γ**), in the NMDA receptor signalling cascade. The downregulation of CaMKII*γ* in the prefrontal cortex results in loss of synaptic plasticity [39]. Dizocilpine, an NMDA receptor antagonist, was shown to simulate this downregulation which could be reversed by pretreatment with antipsychotic drugs like clozapine and haloperidol, thus facilitating NMDA receptor function [39]. Mellios et al. [40] observed that miR-195 is downregulated in postmortem brain of schizophrenia patients. The authors reported that miR-195 targets *brain derived neurotrophic factor* (*BDNF*) expression and indirectly reduces the expression of GABAergic genes, *neuropeptide Y* (*NPY*) and *somatostatin* (*SST*). It is noteworthy that disruption of NMDA-mediated glutamate signaling resulting from dysregulated GABAergic gene expression, has been widely reported in the prefrontal cortex of subjects with schizophrenia [40]. MiR-195 has also been speculated to be the main regulator of the schizophrenia network in partnership with *early growth response 3* (*EGR3*) [41]. Moreover, Beveridge et al. [42] showed significant upregulation of miR-181b in the temporal cortex of postmortem Schizophrenia patients and downregulation of their respective targets such as *calcium sensor gene visinin-like 1* (*VSNL1*) and the *ionotropic AMPA glutamate receptor subunit* (*GRIA2*) [42].

### 2.2. Neurotoxicity as a Result of Aggregation or Accumulation of Toxic Proteins

Aggregation and accumulation of toxic levels of undesirable proteins either due to overexpression or incorrect processing results in death of a targeted group of neurons which manifests symptoms that depict a loss of function of those cells. Such defects result in debilitating neurological diseases like in Parkinson's disease (PD), where *α*-synuclein accumulates in dopaminergic neurons. In the Alzheimer's disease (AD) condition, inappropriate enzymatic activity results in accumulation of A*β* amyloid protein aggregates.

#### 2.2.1. **α*-Synuclein* Accumulation and Parkinson's Disease

Five genes, **α*-synuclein*, *parkin*, *dj-1*, *pink1*, and *lrrk2*, have been implicated to play a role in the pathogenesis of PD [43, 44]. Significantly increased levels of *α*-synuclein are detrimental to dopaminergic neurons [45] and fibrillar *α*-synuclein accumulation in Lewy bodies and Lewy neurites have been reported in postmortem samples [46]. These aggregates display impaired function of chronic loss of dopaminergic neurons in the substantia nigra pars compacta in patients who manifest disabling motor abnormalities accompanied by dementia and hallucinations [45].

Studies on the role of miRNAs in PD started with deletion of *Dicer *in dopamine neurons. Absence of *Dicer* resulted in progressive loss of dopamine neurons as well as expression of Parkinson's-like behaviors [47] and reduced neuronal size and astrogliosis in dopamine-receptive neurons lacking *Dicer* [48]. MiR-7, expressed mainly in the neurons, has been shown to protect the cells against oxidative stress by repressing **α*-synuclein* translation. In both cultured cells and mice model of PD, administration of 1-methyl-4-phenyl-1,2,3,6-tetrahydropyridine (MPTP) resulted in decreased expression of miR-7 and increased **α*-synuclein* expression [45]. Similarly, Doxakis [49] found that both miR-7 and miR-153, which are predominantly expressed in the brain, could repress **α*-synuclein* expression and regulate it posttranscriptionally [49]. Midbrain dopaminergic neurons specific miRNA, miR-133b was found to control the maturation of the dopaminergic neurons by suppressing the homeodomain transcription factor, *Pitx3*. Kim et al. [47] reported that *Pitx3*, which was significantly downregulated in PD brains, was not only a direct target of miR-133b but it could also regulate transcription of miR-133b through a sensitive negative feedback loop [47].

Another group, Asikainen et al. [50] went a step further to focus on other genes as well. Analysis of expression of miRNAs in PD-associated *C. elegans* models showed underexpression of the family of miR-64 and miR-65 in human A53T **α*-synuclein* overexpression and mutated vesicular *catecholamine transporter* (*cat-1*) model animals, as well as underexpression of let-7 family members in the **α*-synuclein* overexpression and *parkin* (*pdr-1*) mutated strains. MiR-64 and miR-65 potentially target *mdl-1* and *ptc-1* genes which were highly expressed in the model animals compared to healthy ones as well as in miR-64/miR-65 knockout animals [50]. However, there is a need to validate these results in rodent experiments as well as postmortem human brain samples.

#### 2.2.2. A*β* Aggregation and Alzheimer's Disease

**Figure 2:**
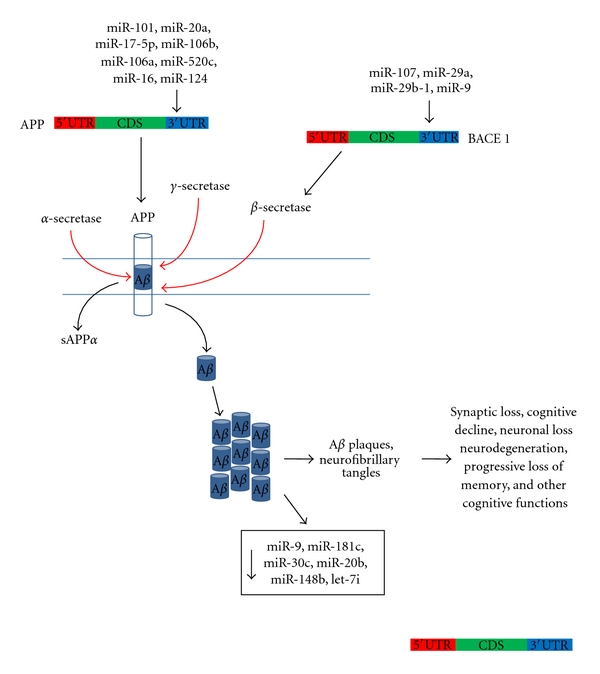
Pathology of Alzheimer's disease and miRNAs involved in its neurotoxicity. There is cooperative regulation of the proteins involved in A*β*-induced neurotoxicity with several miRNAs to fine-tune their expression. Abbreviations: APP: amyloid precursor protein; BACE1: *β*-secretase or *β*-site APP-cleaving enzyme; CDS: coding sequence; sAPP*α*: secretory APP*α*; UTR: untranslated region.

Alzheimer's Disease (AD) is characterized by clinical manifestations of progressive loss of memory and other cognitive functions. Early synaptic loss contribute to disease progression [51] and subsequent neuronal loss leads to generalized brain atrophy. Formation of neurofibrillary tangles (NFTs) that comprise the microtubule associated protein, tau, and neuritic plaques composed of amyloid-*β* (A*β*) are the cause of pathogenesis in AD [52, 53]. A*β* (predominantly 40 amino acid polypeptide, A*β*40) is a naturally occurring protein. It is cleaved from the larger amyloid precursor protein (APP) by the synapse formation regulator enzyme, *α*-secretase [52]. However, proteolytic cleavage by *β*-secretase (BACE-1 or *β*-site APP-cleaving enzyme) and *γ*-secretase results in the formation of another longer form, A*β*42, which forms higher-order aggregates which subsequently result in plaque deposition (Figure 2). Although it is well-established that A*β*42 accumulation gives rise to AD pathology, the mechanism and signalling cascades that give rise to its toxicity have yet to be elucidated [54]. Nevertheless, inflammation, mitochondrial dysfunction, oxidative stress, and calcium dysregulation have been proposed to contribute to the toxicity [55].

Profiling of postmortem human AD brain samples has verified that significant changes in miRNA expression occur in several brain regions [56]. The miRNAs studied included miR-20a family and miR-107 which regulate *APP *and *BACE1,* respectively [57–59]. Schonrock et al. [53] first examined how A*β* itself causes neuronal miRNA deregulation that contribute to the pathological mechanisms of AD. Treatment of primary cultures with A*β* peptides downregulated almost 50% of the analysed miRNAs. Similar results were also demonstrated at the onset of A*β* plaque formation in the A*β*42-depositing APP23 mice. These results showed the downregulation of miR-9, miR-181c, miR-30c, miR-20b, miR-148b, and let-7i as observed in human AD studies [58]. The downregulated miRNAs miR-9, miR-30, and miR-20 were also predicted to affect target genes that were implicated in axonal guidance. Therefore, neuronal miRNA deregulation and consequently, gene dysregulation at the early stages due to A*β* may be an important factor contributing to disease progression and toxicity of AD [53]. Nunez-Iglesias et al. [60] also found that miR-148b, miR-20b, and miR-181c were downregulated among the 48 significantly deregulated miRNAs in the parietal lobe cortex of AD patients.

In AD brain cortical and cortical white matter samples a reduced expression of miR-101 has also been observed [58, 60]. MiR-101 negatively regulates *APP* and hence is considered to have a potential of being developed as a therapeutic target to attenuate A*β*42 accumulation and downstream pathogenic mechanisms underlying AD [61]. Moreover, miR-20a, miR-17-5p, and miR-106b (miR-20a family), miR-106a, miR-520c, and miR-16 have also been shown to regulate *APP* expression [59, 62, 63]. Smith et al. [64] found that miRNAs regulate neuronal splicing of *APP in vivo. *MiR-124 was considered to serve as an indirect regulator of *APP* splicing [64].

Apart from APP, the enzyme responsible for inappropriate cleavage of APP, BACE1 has also been studied for variations that might give rise to the AD disease. MiRNA expression studies on human brain tissue showed significantly reduced miR-107 levels in patients in the early stages of the disease [57]. This miRNA was validated to target the 3′UTR of *BACE1* and hence, could have a crucial role to play in accelerated disease progression through regulation of BACE1. MiRNA profiling showed that the miR-29a/b-1 cluster was significantly decreased in AD patients presenting abnormally high BACE1 protein and miR-29a, miR-29b-1, and miR-9 were observed to negatively regulate *BACE1* expression in primary cell culture. Loss of specific miRNAs like the miRNA cluster miR-29a/b-1 in sporadic AD was therefore considered to contribute to increased BACE1 and subsequently A*β* levels [58].

The development and progression of AD is due to elevated inflammatory signals triggered by overactivation of NF*κ*B. This transcription factor specifically upregulated miR-146a in the AD brain, and negatively regulated an important repressor of the brain inflammatory response, *complement factor H* (*CFH*) [65]. This correlated with downregulation of *CFH* as well as *interleukin-1*β** and *A*β*42* in oxidatively stressed human neural (HN) primary culture cells. Hence, NF-*κ*B sensitive miRNA-146a-mediated modulation of *CFH* gene expression could also regulate an inflammatory response in AD brain. MiRNA-146a has also been shown to be an NF-*κ*B-sensitive endotoxin-responsive gene, and predicted to target *tumor necrosis factor receptor-associated factor 6* and *IL-1 receptor-associated kinase 1* mRNAs [66, 67].

Elevated expression of miRNA-146a correlated with senile plaque density and synaptic pathology as well as pathophysiological stress factors *in vivo* and *in vitro* as well as in AD postmortem brain samples [68]. Elevated levels of the *BACE1*-antisense transcript alongside dysregulated miR-485-5p levels in AD patients implicating increased stability of the *BACE1* transcript by preventing interaction between miR-485-5p and its seed sequence [69].

These studies confirm that the accumulation of APP and subsequent A*β* by-products lead to neurotoxicity that manifests as Alzheimer's disease. This is probably mediated by a network of miRNAs, in particular miR-9, miR-30 and miR-20, miR-29a/b-1 cluster, miR-146a, miR-124, and miR-485-5p. These miRNAs have been demonstrated to either target the *APP* splicing/expression or the production of *A*β**.

## 3. Genetically Transferred (Inherited) Cause of Neurotoxicity

Genetic inheritance of genes implicated in toxicity or polymorphisms are also risk factors of neurotoxicity and could have dire consequences as compared to sporadic onset. Genetic multiplication of the implicated genes like *α*-synuclein in PD results in early onset and increasing severity of dementia in a gene dosage-dependent manner [70, 71].

Single nucleotide polymorphism genotyping identified *fibroblast growth factor 20 *(*FGF20*) at chromosome 8p21.3–22 as a risk factor for PD [72]. The strongest association was observed between single-nucleotide polymorphism at rs12720208 in the 3′UTR of *FGF20*. The risk allele was shown to disrupt (mutate) the binding site for miR-433, increasing translation of *FGF20* that was accompanied by an increase in *α*-synuclein expression, in both *in vitro* and *in vivo* studies. Hence, single-nucleotide polymorphism of the *FGF20* gene resulted in chronic elevation of *α*-synuclein levels in human brain that translated to increased susceptibility to PD. It is noteworthy that early in life, FGF20 is beneficial to proliferation, differentiation, and even neuroprotection of the midbrain dopaminergic neurons. However, at later stages of development, the significantly elevated levels of FGF20 could indirectly contribute to neurotoxicity that results in dopaminergic neuron death. This risk allele is thus an important component to determine individual susceptibility to this debilitating disease and has opened the way for the potential use of miR-433 and FGF20 as therapeutic and diagnostic markers [72].

Huntington disease (HD) is largely a hereditary neurodegenerative disorder associated with expansion of the polyglutamine region in the gene encoding the protein huntingtin (Htt). Neurodegeneration results in defects in cognition and motor control, leading to chronic loss of cortical and striatal neurons and death. The mutant Htt confers toxicity to the neurons and eliminates the neuroprotective effects of the wild-type Htt [73]. In normal neurons, transcriptional repressor, REST (repressor element 1 silencing transcription factor) regulates the expression of Htt [74]. Mutant Htt showed reduced expression of proteins that were vital for neuronal survival and function, conferring neurotoxicity. Johnson and Buckley [75] examined the abnormal expression of neuron-specific miRNAs in the tissues from HD patients and observed significant downregulation of miR-132. MiR-132 was shown to target *REST*, that was required for neurite growth and could therefore, account for the loss of signal transduction of the diseased neurons [76–78]. MiR-34b was observed to be stable in plasma and significantly elevated in HD gene-carriers even before the symptoms were presented [79]. Significant downregulation of numerous miRNAs was observed in cortices of HD patients, inclusive of the bifunctional brain enriched miR-9 and miR-9* which targeted *REST* and *CoREST*, making up the REST silencing complex [80].

MiRNAs implicated in neurotoxicity in diseases of CNS and their related functions are listed in Table 1.

## 4. Brain Tumour(s) Induced Neurotoxicity

Dysregulation of gene expression within the neurons could also result in uncontrolled cell growth leading to formation of tumours. Brain tumours are categorized as glioblastoma in adults and medulloblastoma in children. Glioblastoma is the most common and most lethal brain tumour. MiR-221 and miR-222 are overexpressed in human glioblastoma. These miRNAs have been validated to negatively regulate the *protein tyrosine phosphatase *μ** (*PTP*μ**) gene that has been observed to be downregulated in these tumour cells [96]. The speculated inverse relationship was observed *in vitro* as well as in glioma cancer patient samples.

Inhibition of miR-10b decreased growth of the tumour by retraction from the cell cycle and encouraging programmed cell death [97]. This was facilitated by expressing the targets of miR-10b—*BCL2L11/Bim*, *TFAP2C/AP-2*γ**, *CDKN1A/p21*, and *CDKN2A/p16* that regulated controlled cell growth. MiR-10b was therefore responsible for uncontrolled cellular growth by downregulating proapoptotic genes. Decreased survival of glioblastoma patients with high miR-10b expression was observed, implicating its *in vivo* functions as well. Successful treatment was also reported to suggest its therapeutic potential [97].

MiRNA-146a was shown to be upregulated in epidermal growth factor receptor (EGFR) dysregulated cells in gliomas. MiR-146a targets *Notch1* which maintains neural stem cells, thus controlling proliferation and differentiation of neural stem cells. In gliomas, upregulation of miR-146a was proposed to counter tumourigenicity, in a negative feedback-loop fashion [98]. MiR-21, an antiapoptotic miRNA has been widely implicated in cancer. It was found to be upregulated in medulloblastoma and inversely correlated to the metastasis suppressor *PDCD4*. PDCD4 positively regulated E-cadherin and tissue inhibitor of metalloproteinase-2 (TIMP2), the negative modulators of cancer cell migration, thus resulting in an increased cell motility and migration [99]. *LRRC4* is a glioblastoma suppressor gene specific to the brain and a target of miR-381. Repression of *LRRC4* by miR-381 promoted glioma cell proliferation. At the same time, LRRC4 has also been found to inhibit the expression of miR-381 in the cell and decrease cell proliferation and tumour growth [100]. In addition, a recent study by Srinivasan et al. [101] identified ten miRNAs that correlated to the survival of glioblastoma patients. Three miRNAs, miR-20a, miR-106a, and miR-17-5p, were protective in nature and seven miRNAs, miR-31, miR-222, miR-148a, miR-221, miR-146b, miR-200b, and miR-193a, were categorized as risk markers in terms of patient survival. Protective miRNAs (decreasing tumorigenicity) were more abundant in the low risk group while the miRNAs that increased tumorigenicity (risk indicators) were more abundant in the high-risk group [101]. Such information is thus vital in prognosis of the disease.

## 5. Exogenous Factors Contributing to Neurotoxicity

### 5.1. Alcohol

Long-term ethanol abuse could lead to ethanol-induced neurotoxicity which changes the expression of genes implicated in myelination, ubiquitination, apoptosis, cell adhesion, neurogenesis, and neural disease [102]. Gene expression profiling of postmortem brain of long-term alcohol abusers allowed determination of the effects of alcohol in the brain [102–107]. Examination of dysregulated miRNAs in the samples of alcoholic and nonalcoholic age- and sex-matched controls showed that expression of approximately 35 miRNAs was significantly upregulated in the alcoholic group [108]. Target prediction analysis showed an inverse relationship between the upregulated miRNAs and the targeted mRNA in human alcoholic cases. These mRNAs and their genes were implicated to downregulate the pathways involved in the central nervous system development and synapse formation [109].

*Peripheral myelin protein 22* (*PMP22*) is regulated by long term ethanol use and a target of miR-29a thus suggesting that miRNAs could regulate myelin gene expression [102, 104, 106, 110]. Moreover, miRNAs altered in neurodegenerative diseases like Alzheimer's, Parkinson's, and prion diseases have also been shown to be significantly dysregulated in the prefrontal cortex of alcoholics [45, 59, 102, 111].

Pietryzkowski et al. [112] showed that the *large-conductance calcium-and voltage-activated potassium channel* (*BK*) was a target for miR-9 and exposure to alcohol upregulates miR-9 and mediated posttranscriptional reorganization in *BK* mRNA splice variants. In a study to demonstrate how the neurons adapt or confer protection during acohol exposure, the authors showed that an increase of miR-9 correlated to altered expression of alpha subunit of *BK* channel isoforms [112]. Notably, the BK channel isoforms, confered varied sensitivity to ethanol exposure. Some isoforms were very sensitive while others had low sensitivity or even innate tolerance to ethanol. Pietryzkowski et al. [112] showed that 3′UTR of *BK* isoforms of high ethanol sensitivity had a miR-9 binding site that was absent in the ethanol-tolerant BK isoforms. Thus, the expression of the ethanol insensitive *BK* isoforms was not affected by ethanol. Hence, tilting the homeostasis of BK isoforms towards the ethanol-tolerant isoforms in the brain to maintain the expression of BK for neuronal plasticity and function [102, 105–107, 113, 114].

Experiments using cell culture models of the second trimester fetal neuroepithelium showed that fetal stem cells (NSCs)/progenitor cells (NPCs) were a direct target of ethanol [115]. It has been hypothesized that ethanol promotes cell cycle, resulting in increased maturation, and consequently, depletion of stem and early progenitor cells [115, 116]. Additionally, differentiating neuroblasts, derived from ethanol preexposed neurosphere cultures exhibited significantly increased migration, compared to nonexposed controls [117] which supported continual organizational effects of ethanol in NSCs/NPCs [116]. Four miRNAs, miR-9, miR-21, miR-153, and miR-335 had been shown to be suppressed by ethanol in NSCs/NPCs [118]. Ethanol mediated simultaneous suppression of miR-21, miR-153, and miR-335 accounted for the resistance of ethanol-exposed NSCs/NPCs to apoptosis. MiR-335 suppression was suggested to be the cause of ethanol-induced cell proliferation in neurosphere cultures [116]. Notch receptor ligand, *Jagged-1*, and neuron-specific RNA binding protein *ELAVL2/HuB* were both predicted targets of at least three of the four suppressed miRNAs, with miRNAs-335, -21, and -153 targeting *Jagged-1* and miRNAs-335, -153, and -9 targeting *ELAVL2, *and the expression of both genes was thereby induced by ethanol in neurosphere cultures [118]. *ELAVL2/HuB* overexpression promoted neuronal differentiation [119] and *Jagged-1*-induced proliferation established neuronal identity [120]. Both these processes of proliferation and differentiation being triggered simultaneously by ethanol would account for promotion of NSC maturation and cell cycle induction without cell death via derepression of miRNA-inhibited neuronal identity factors [118]. The upset of the balance between cell survival and proliferation depletes stem cells contributing to dysregulation of normal function, and thus neurotoxicity in the growing fetus. Hence, excessive ethanol consumption during pregnancy could lead to growth retardation, mental retardation, and a mix of craniofacial, cardiovascular and skeletal defects collectively termed the “fetal alcohol syndrome” or FAS [121]. MiRNA expression was sensitive to ethanol especially during development and could mediate ethanol teratology [118, 122]. Prenatal ethanol exposure resulted in upregulation of miR-10a and downregulated *Hoxa1* expression in fetal brains [122]. In cultured embryos, dysregulation of *Hoxa1* gene lead to birth defects especially in the brain [122]. The group also established that folic acid could rescue this ethanol-induced teratogenesis by downregulation of miR-10a expression [122].

### 5.2. Nicotine

There is a high correlation between alcohol consumption and tobacco use. Both preclinical and clinical data provide evidence that nicotine administration increases alcohol intake and nonspecific nicotinic receptor antagonists reduce alcohol-mediated behaviors [123].

Nicotine-mediated neurotoxicity is well established and chronic use of nicotine confers addiction and altered neuronal functions. Huang and Li [124] tested the effects of nicotine on neuronal cultures. They reported that short-term nicotine exposure upregulated the expression of 11 miRNAs (miR-188, miR-137, miR-328, miR-181b, miR-503, miR-140*, miR-351, miR-125b, miR-93, miR-26a, and miR-25), while downregulating the expression of an additional 14 miRNAs (miR-301a, miR-10b, miR-30a-5p, miR-186, miR-29c, miR-101a, miR-152, miR-21, miR-30c, miR-374, miR-335, miR-210, miR-98, and miR-352). MiR-140* was upregulated in nicotine exposure and targeted several genes implicated in neuronal function, including *Dnm1* which encodes a large GTPase, *dynamin-1*, required for synaptic endocytosis [124, 125]. Therefore, nicotine-induced neural activities could be modulated by miR-140*. Furthermore, morphine-administered rats also revealed an enriched postsynaptic localization of dynamin 1 in the hippocampus [126], demonstrating a potential role of dynamin 1 and miR-140* in drug-induced neural plasticity and subsequent neurotoxicity.

### 5.3. Morphine

**Figure 3:**
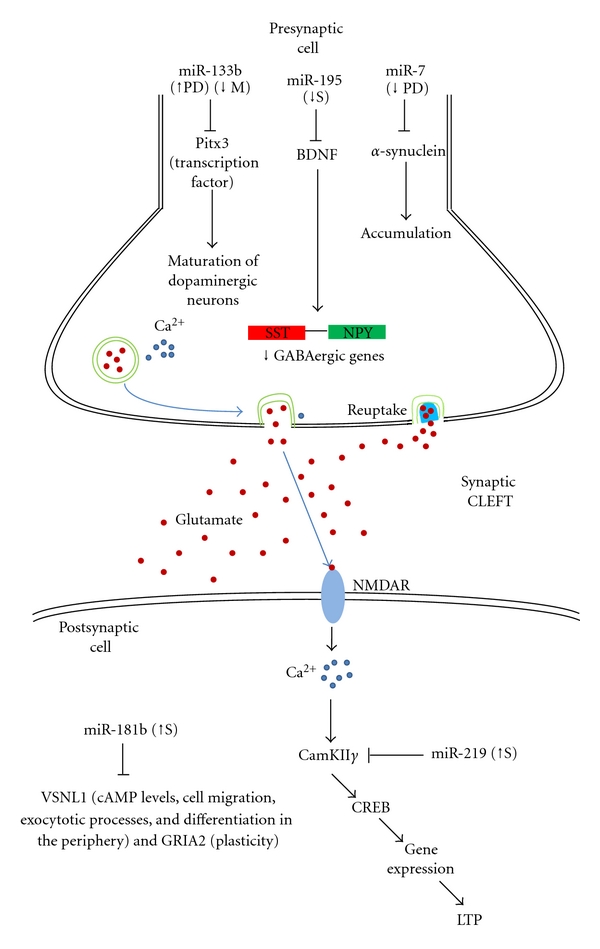
Overview of some miRNAs implicated in cellular dysfunction leading to neurotoxicity and manifesting as neurodiseases. Abbreviations: BDNF: brain-derived neurotrophic factor; CaMKII*γ*: Ca^2+^/calmodulin-dependent kinase II *γ*; CREB: cAMP response element binding protein; GRIA2: ionotropic AMPA glutamate receptor subunit; LTP: long-term potentiation; M: morphine; NMDAR: N-methyl-D-aspartate receptor; NPY: neuropeptide Y; PD: Parkinson's disease; S: schizophrenia; SST: somatostatin; VSNL1: calcium sensor gene visinin-like 1.

Opioid drugs, such as morphine, are a class of powerful analgesics that are used for treating many forms of acute and chronic pain. Their chronic use results in undesirable effects such as drug tolerance, opioid-induced pain, and opioid dependence as well as reducing the size of the dopaminergic neurons [127, 128]. This analgesics also forms a common drug of abuse that could have dire consequences for the neurons upon repeated intake. Morphine functions via the *μ* opioid receptor to bring about changes in miRNA expression in neurons. Morphine regulated the miR-133b : *Pitx3* pair to increase *Pitx3* expression in immature hippocampal neurons, thus promoting neurotoxicity in neuronal differentiation [129]. Pitx3 was responsible for activation of the dopaminergic neuron gene expression and function as discussed in toxicity leading to PD, both of which share similar gene dysregulation. It is likely that this same mechanism of toxicity could result in morphine abusers developing symptoms similar to PD patients (Figure 3). Also, morphine has been shown to elevate let-7 expression which targets the **μ* opioid receptor*, thereby decreasing protein levels and sensitivity to the drug, giving rise, to drug tolerance.

### 5.4. Cocaine

Cocaine is a strong stimulant of the central nervous system that increases levels of dopamine, and results in accumulation of this neurostimulant in the neurons. Cocaine is another drug of abuse that results in increased craving for the drug over long period of consumption [127]. Changes in neuronal networks form the basis of decreased responses to the same dose of a drug over time [130]. MiR-212 has been shown to be highly expressed in the striatum of rats. Hollander et al. [131] showed that increased miR-212 expression correlated with prolonged consumption of cocaine. MiR-212 has been demonstrated to decrease the downstream signalling response of cocaine by increasing sensitivity of adenylyl cyclase, thereby, magnifying the stimulatory effects of the drug on cAMP response element binding protein (CREB) signalling [131]. Im et al. [132] have also attributed miR-212 interaction with the X-linked transcriptional repressor *methyl CpG binding protein 2* (*MeCP2*) to decrease expression of MeCP2 and downstream *BDNF* which is responsible for the plasticity induced in striatal neurons resulting in cocaine addiction [132]. This is yet another example of fine-tuning in the central nervous system in response to adverse reactions like addiction in an attempt to counter it. Cocaine-induced neurotoxicity thus could be reversed in the presence of miR-212.

MicroRNAs regulate the expression of drug-metabolizing enzymes and transporters. Brain specific miR-124a was found to be downregulated by psychoactive drugs like cocaine, methadone, and fluoxetine in BE(2)-M17 and SH-SY5Y cells [133]. MiRNA-18a was also found to be downregulated in the presence of DMT, 5-MeO-DMT, harmaline, methylphenidate, and methadone (psychoactive drugs). MiR-18a has been shown to regulate posttranscriptional gene expression of *glucocorticoid receptor* (*GR/NR3C1*) [134] and *estrogen receptor-alpha* (*ERa/NR3A1*) [135, 136] which are ligand inducible transcription factors controlling development, metabolism, immune response, and neuronal differentiation [133]. Interestingly, miR-18a was elevated by desipramine, a tricyclic antidepressant. Prolonged treatment with desipramine increased miR-18a expression while downregulating the expression of *GR/NR3C1* in Wistar-Kyoto rats and manifested into depressive behavior. Identification of the underlying mechanisms would ultimately provide increased understanding of the effects of the drugs and cellular defense against xenobiotic agents. Therefore, psychoactive agents significantly alter the expression of neuronal miRNAs.

### 5.5. Prion as Biological Neurotoxins

Prion disease or transmissible spongiform encephalopathies (TSEs) are the consequence of infection that results in a fatal structural change of the normal cellular prion protein, PrPC (prion protein cellular) into PrPSc (prion protein scrapie) [137]. Upregulation of miR-342-3p has been observed in bovine spongiform encephalopathy infected macaques and correlated to increased expression in brain samples of (sporadic) human patients [111]. Similarly, using mouse model of prion disease, Saba et al. [138] identified a group of 15 miRNAs to be dysregulated. Besides the miR-342-3p, the expression of miR-320, let-7b, miR-328, miR-128, miR-139-5p, and miR-146a were also found to be upregulated and miR-338-3p and miR-337-3p were downregulated in the prion induced neuronal toxicity [138]. The authors proposed that the coordinated dysregulation of these miRNAs could be a consequence of abnormal accumulation of PrPSc that resulted in neurotoxicity. The process could include compensatory modulation of miRNA expressions that regulated the neuronal activities as well as protein degradation and signaling pathways that could have led to failure in neuronal function.

Other exogeneous agents comprising environmental stressors and toxic compounds like hexahydro-1,3,5-trinitro-1,3,5-triazine (RDX) could also cause dysregulation in miRNA profile that results in neurotoxicity [139].

## 6. Brain Injury-Induced Neurotoxicity

Generally, brain injury in any form triggers the accumulation of substances (neurotransmitters, ions, fluid, etc.) that are initially produced to compensate or repair the damage. However, uncontrolled accumulation of these substances will lead to neurotoxicity in the CNS. Injury to the brain could be caused by endogenous factors such as ischemia due to embolism/thromboembolism of an artery or tumour growth that results in anoxia and glucose deficiency to the neurons and other cells in the brain. Physical injury to the brain could also cause trauma that confers neurotoxicity.

### 6.1. Cerebral Ischemia

Cerebral ischemia is an event that leads to neurotoxicity during the onset of ischemia as well as during reperfusion. Temporal regulation of miRNA expression has been shown in the brain of rat models subjected to middle cerebral artery occlusion (MCAo) [140]. In addition, biphasic expression of miRNAs has also been demonstrated in the same animal models. The temporal and biphasic regulation of these miRNAs have been proposed to play a crucial role either in the acute injury phase or the late recovery phase [141]. An upregulation of the antiapoptotic miR-21 has been shown to protect neurons from death in cerebral ischemic model. MiR-21 was demonstrated to target *FASLG*, a member of the tumour necrosis factor-*α* family and cell death inducing ligand [142]. In an *in vitro* model of cerebral ischemia, increase of miR-497 expression following oxygen-glucose deprivation, correlated with increased cell death and downregulation of its antiapoptotic proteins, *Bcl2* and *Bclw* [143].

A vital new discovery of neurotoxicity is the contribution of postinjury edema that results in accumulation of water. Transport of water in and out of brain cells are controlled by aquaporins that are located at the cell membrane. The edema formation induces death of neurons, and hence neurotoxicity, which can be countered by controlling edema formation. *AQP4* and *AQP1* are expressed predominantly in astrocytes and is crucial in fluid clearance in cerebral edema [144]. Sepramaniam et al. [145] investigated changes in aquaporin 1 and 4 expression in the MCAo rat brain and identified miR-320a as a potential negative modulator of *AQP1* and *AQP4*. Anti-miR-320a could reduce infarct volume in cerebral ischemia with concurrent elevation in *AQP1* and *AQP4* mRNA and protein expression. Inhibition of miR-320a subsequently reduced the infarct volume and improved the neurological functions of the rat models. Similarly, downregulation of miR-320 was also observed in peripheral blood of stroke patient who showed good recovery and good clinical outcome (modified Rankin Scale, mRS ≤ 2) as compared to patients with poor clinical outcome (mRS > 2) [146].

### 6.2. Traumatic Brain Injury

Altered expression of miRNAs in the cortex and hippocampus was also observed in traumatic brain injury models [147]. Differential regulation of several miRNAs (miR-107, miR-130a, miR-223, miR-292-5p, miR-433-3p, miR-451, miR-541, and miR-711) was observed with controlled cortical impact injury [148]. These miRNAs were predicted to regulate the cellular processes that comprised differentiation, proliferation, signal transduction, and transcriptional regulation [148]. It is noteworthy that miR-107 that was downregulated in traumatic brain injury inversely regulated progranulin which was involved in wound repair or healing [149, 150]. Thus, in brain injury or during ischemia, the consequences of neurotoxicity are compensated (remedial activities) almost immediately and are controlled by the riboregulators (miRNA) as the first level of regulation.

## 7. Conclusion

MiRNAs have been found to be highly dysregulated in different stages of neurotoxicity. Neurotoxicity ranges from the impact of drugs to neurodegeneration in cells of the nervous system. These processes have been found to result in changes in expression of specific miRNAs. Hence such miRNAs could be exploited as potential biomarkers for diagnostic or prognostic purposes. Moreover some of these miRNAs can be developed as therapeutic agents or targets to prevent neurotoxicity.

## Figures and Tables

**Table 1:** MicroRNAs involved in diseases due to dysregulation of normal cellular activites.

Description	Upregulated (miRNA)	Downregulated (miRNA)	Function/target
Dysregulation of NMDA receptor function			

Schizophrenia		miR-219	Targets CamKII*γ* to result in NMDA receptor hypofunction [39]
	miR-181b Other dysregulated miRNAs: miR-199a, miR-128a, and miR-128b [81–83]		VSNL1 and GRIA2 [42]

Aggregation and accumulation of toxic proteins			

*α*-synuclein in dopaminergic neurons: Parkinson's disease		miR-7 [45]	*α*-synuclein
		miR-153 [49]	*α*-synuclein
		miR-133b [47]	Pitx3
		miR-64 and miR-65 [50]	mdl-1 and ptc-1

A*β* aggregation in basal forebrain, hippocampus, and association cortices: Alzheimer's disease		miR-101	APP [84]
		miR-107	BACE1 [57]
		miR-29a/b-1	BACE1 [58]
		miR-146a	Targets CFH to elicit inflammatory response [85]
		miR-485-5p	Stability of BACE1-antisense prevents repression of BACE1 by blocking this binding site [69]

Inherited disorders			

Parkinson's disease			Risk allele disrupts binding site of miR-433 in 3′UTR of FGF20 [72]
Huntington's disease		miR-132 [76–78]	
		miR-9 and miR-9*	Target REST and CoREST which repress genes vital to neuronal survival and function [80]
Tourette's syndrome	Mutation in miR-189 binding site		SLITRK1 [86]
Williams syndrome	miR-134		LimK1 [24]
Rett syndrome	miR-132		MeCP2 [87, 88]
Fragile X mental retardation	miR-125b		NR2A [89]

Others			

Amyotrophic lateral sclerosis (ALS)		miR-206	Derepresses histone deacetylase 4, an inhibitor of muscle reinnervation [90]
Spinal motor neuron disease		miR-9	Targets NEFH, heavy neurofilament subunit of upper and lower motor neurons, leading to paralysis and death [87]
Spinocerebellar ataxia 1 (SCA1)	miR-19, miR-101,		ATXN1 [84]
	miR-130		
Spinal cerebellar ataxia type 3 (SCA3)		Bantam	Ataxin-3 toxicity, polyglutamine- and tau-induced neurodegeneration [91]
Dentatorubral-pallidoluysian atrophy (DRPLA)		miR-8	Atrophin-1 [92]
Frontotemporal dementia	miR-29b		Downregulation of the secreted glycoprotein, human progranulin [93]
Aicardi-Goutières syndrome			Silencing of RNAse activity leading to miRNA overload [94]
Ageing (ad libitum versus to calorie-restricted diet)	miR-181a-1*, miR-30e and miR-34a in *ad libitum* mice as compared to CR regimen		Target Bcl-2 to increase proapoptosome specific proteins and thus rate of neuronal apoptosis [95]
